# Context dependent role of miR-486 promoting neuroregeneration of primary sensory neurons downstream of interleukin-6 signal transducer

**DOI:** 10.1016/j.omtn.2025.102670

**Published:** 2025-08-06

**Authors:** Theodora Kalpachidou, Kai Kummer, Valentina Handle, David Zimmermann, Maria Peteinareli, Serena Quarta, Natalia Mach, Laura Castaldi, Paul A. Heppenstall, Rainer V. Haberberger, Hermona Soreq, Michaela Kress

**Affiliations:** 1 Institute of Physiology, Medical University of Innsbruck, Schöpfstrasse 41, Innsbruck 6020, Austria; 2 Health University of Applied Sciences Tyrol / FH Gesundheit Tirol, Innrain 98, Innsbruck 6020, Austria; 3 Epigenetics and Neurobiology Unit, EMBL Rome, Via Ramarini 32, Monterotondo 00015, Italy; 4 Neuroscience Area, International School for Advanced Studies (SISSA/ISAS), Via Bonomea 265, Trieste 34136, Italy; 5 Department of Anatomy and Pathology, School of Biomedicine, The University of Adelaide, Adelaide, SA 5005, Australia; 6 The Alexander Silberman Institute of Life Sciences, the Edmond and Lily Safra Center for Brain Sciences, and Department of Biological Chemistry, The Hebrew University of Jerusalem, Edmond J. Safra Campus, Givat Ram, Jerusalem 9190401, Israel

**Keywords:** MT: Non-coding RNAs, IL-6, IL6ST/gp130, gp130, miRNAs, mmu-miR-486-5p, peripheral nerve injury, neuropathic pain, neuronal regeneration

## Abstract

The pro-inflammatory cytokine interleukin-6 (IL-6) via its IL-6 signal transducer (IL6ST/gp130) plays an important role in neuronal survival, neuro-regeneration, and pathological pain. While its critical importance in the nervous system is well established, the underlying molecular mechanisms and the involvement of microRNAs (miRNAs) as critical regulators of biological processes in health and disease are not sufficiently understood. We identified miR-486-5p as the single significantly deregulated miRNA in sensory neurons with a conditional depletion of gp130. *In situ* hybridization and immunofluorescence in dorsal root ganglia (DRG) localized miR-486 to small diameter neurons, including peptidergic nociceptors. miR-486^−/−^ mice exhibited normal baseline and neuropathic pain-like behaviors and recovered similarly to wild-type (WT) littermate controls in response to sciatic crush injury. On the other hand, DRG neurons derived from mice with a conditional deletion of IL6ST/gp130 in Na_v_1.8-expressing primary afferent nociceptors (SNS-gp130^−/−^) show strongly compromised neuro-regeneration, which was significantly rescued by overexpressing miR-486, indicative of a specific role of miR-486 in IL-6/gp130-dependent neuro-regenerative processes. Our findings highlight context-dependent differential expression and roles of miRNAs after nerve injury driving nerve regeneration versus neuropathic pain.

## Graphical abstract

**Figure:**
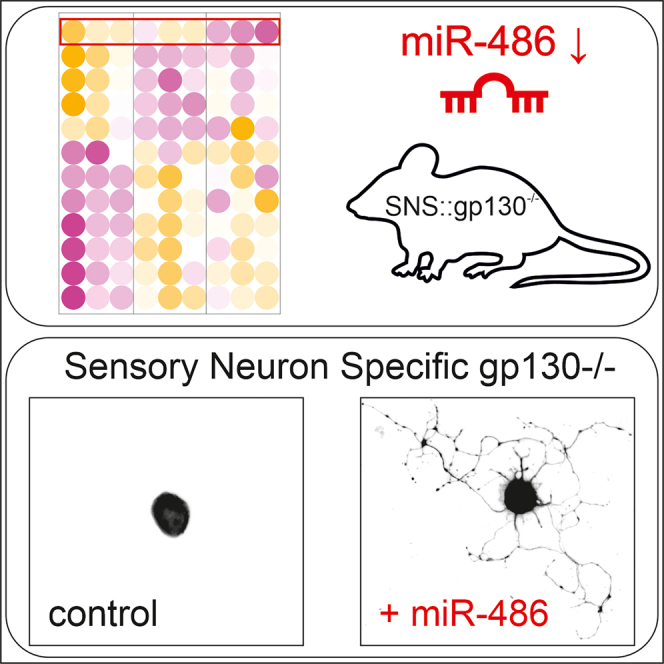

## Teaser

Kalpachidou, Kress, and colleagues identify miR-486 as a miRNA promoting neuronal regeneration. Their findings uncover a novel, context-dependent role for miR-486 and identify molecular pathways involved in IL-6/gp130-induced support of peripheral nerve regenerative processes.

## Introduction

The classical proinflammatory cytokine interleukin-6 (IL-6) is critically involved in the development, differentiation, and function of neurons.^1^^,^^2^ Immune cells including macrophages, glial cells as well as neurons synthesize and secrete IL-6 (reviewed in studies by Juttler et al.^3^ and Scholz and Woolf^4^). IL-6 levels in the adult peripheral nervous system (PNS) are low; however, IL-6 levels dramatically increase following peripheral nerve injuries, particularly at the injury site and likely due to upregulated synthesis in Schwann cells, neurons, and invading immune cells.^5^^,^^6^^,^^7^^,^^8^ IL-6-like cytokines sensitize nociceptors and control neuronal excitability, ion channel expression and function as well as responsiveness to thermal and mechanical stimuli.^9^^,^^10^^,^^11^^,^^12^^,^^13^^,^^14^^,^^15^^,^^16^^,^^17^^,^^18^^,^^19^^,^^20^ Furthermore, mice with a global depletion of IL-6 exhibit reduced mechanical hypersensitivity in response to nerve injury.^21^ Additionally, IL-6 can exert completely opposing effects by supporting neuronal survival after injury or causing neuronal degeneration and cell death.^22^ It acts through the IL-6 signal transducer gp130 (i.e., IL6ST), which is shared by a large family of IL-6 like cytokines.^23^ Notably, gp130 can be activated by the membrane-bound (mIL-6R) or the soluble ligand-binding IL-6 alpha-receptor (sIL-6R) subunits, and the IL-6/IL-6R/gp130 heteromeric complex initiates the classical JAK/STAT signaling cascade to regulate inflammatory processes, neuronal function, and neuro-regeneration.^15^^,^^16^^,^^17^^,^^23^^,^^24^^,^^25^ Moreover, signaling via gp130 is of critical importance for the development and maintenance of pathological pain^1^^,^^26^^,^^27^ and mice with a conditional deletion of IL6ST/gp130 in Na_v_1.8-expressing primary afferent neurons (i.e., sensory neuron-specific SNS-gp130^−/−^), which are considered nociceptors in the dorsal root ganglia (DRG),^28^ are largely protected from mechanical and thermal hypersensitivity in models of neuropathic and inflammatory pain.^14^^,^^15^^,^^16^^,^^17^^,^^29^ Together, these reports support an overall pain-promoting contribution of IL-6 in neuropathic pain disorders. Beneficial effects of IL-6 include the promotion of neuronal regeneration after injury.^30^^,^^31^^,^^32^ In animal models, deficiency of IL-6 or gp130 leads to impaired axonal regeneration and its important neuroprotective, pro-regenerative role involves an essential crosstalk between nerve growth factor (NGF) and IL-6/gp130 signaling cascades.^11^^,^^12^^,^^13^^,^^15^ Nevertheless, the precise mechanisms for the pro-regenerative action of IL-6 are not sufficiently understood.

Recently, a particular group of microRNAs (miRNAs), including miR-21, miR-431, and miR-511, all of which are controlled by IL-6 in sensory neurons, offer new potential for IL-6-mediated mechanistic insights.^10^ miRNAs are small non-coding RNA species of approximately 22 nucleotides in length that can regulate the expression of entire gene sets.^33^ They are increasingly emerging as relevant master switches involved in the pathogenesis of a plethora of physical and mental disorders.^34^^,^^35^^,^^36^^,^^37^^,^^38^^,^^39^^,^^40^^,^^41^^,^^42^^,^^43^ Specific miRNAs, such as miR-26, miR-155, or miR-365, inhibit IL-6 formation and consequently downstream signaling processes.^44^ However, miRNAs that are generated downstream of gp130 activation have not been sufficiently investigated.

To specifically address the role of IL6ST/gp130 regulated miRNAs in primary afferent nociceptors, we performed a microarray analysis, which revealed that mmu-miR-486-5p (termed miR-486 throughout the manuscript) was the only significantly deregulated miRNA in the DRG of naive SNS-gp130^−/−^ mice. We found miR-486 to be expressed in primary sensory afferents, and therefore assessed its potential contribution to gp130-mediated alterations in nociception, sensorimotor coordination and neuro-regeneration *in vivo* and *in vitro*.

## Results

### miR-486 is expressed in sensory neurons and downregulated in SNS-gp130^−/−^ mice

**Figure 1:**
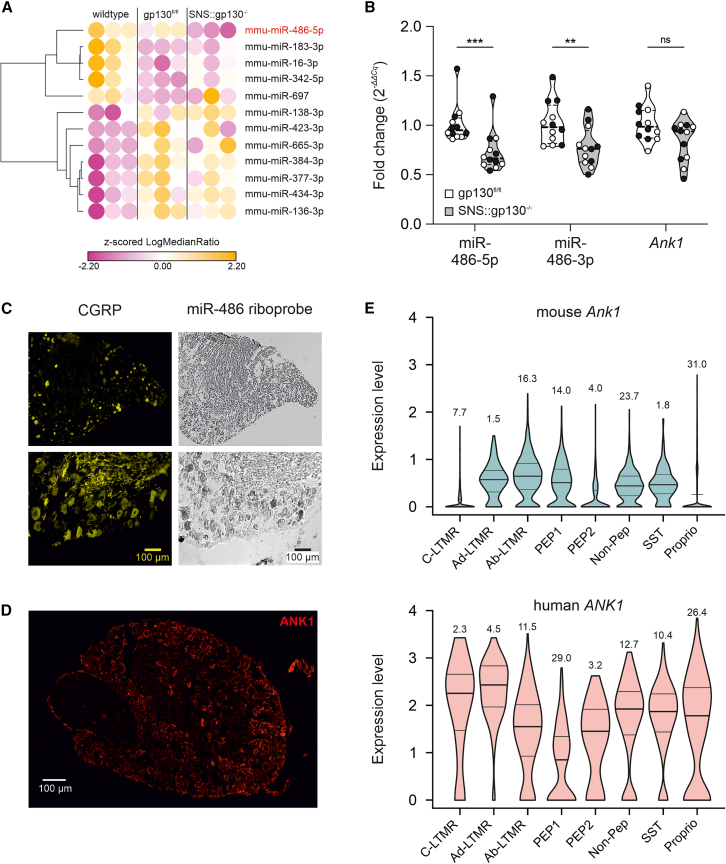
miR-486 is expressed in DRG neurons and downregulated in DRG of SNS-gp130^−/−^ mice (A) Heatmap diagram showing the two-way hierarchical clustering of miRNAs and samples. Each row represents a miRNA and each column represents a sample. The miRNA clustering tree is shown on the left. The color scale shown at the bottom illustrates the relative expression level of a miRNA across all samples: yellow color represents an expression level above mean, pink color represents expression lower than the mean. (B) RT-qPCR demonstrating reduced miR-486, but not *Ank1*, levels in the DRG of SNS-gp130^−/−^ mice. (C) Photomicrographs of i*n situ* hybridization experiments demonstrating that miR-486 expression is expressed in DRG neurons, including CGRP^+^ cells. (D) ANK1 protein expression in WT mouse DRG. (E) *Ank1* expression in mouse^45^ (top) and *ANK1* expression in human^46^ (bottom); naive DRG neurons split into identified neuronal subtypes. C-LTMR, Fam19a4^+^/Th^+^ C-fiber low threshold mechanoreceptors; Ad-LTMR, Aδ low threshold mechanoreceptors; Ab-LTMR, Aβ low threshold mechanoreceptors; PEP1, Tac1^+^/Gpx3^+^ peptidergic nociceptors; PEP2, Tac1^+^/Hpca^+^ peptidergic neurons; Non-Pep, non-peptidergic neurons; SST, somatostatin positive pruriceptors; Proprio, proprioceptors. Numbers above violin plots indicate percentage of cells expressing *Ank1* for mouse and *ANK1* for human DRG neurons; scale bar 100 µm; In panels (B) and (E) data are shown as violin plots with medians (solid lines) and interquartile ranges (dotted lines); ∗∗∗*p* < 0.001 and ∗∗*p* < 0.01; ns, not significant.

We hypothesized that miRNAs are regulatory hubs in the molecular processes underlying deficits in somato-sensation, neuropathic pain, and neuro-regeneration of SNS-gp130^−/−^ mice.^14^^,^^15^^,^^16^^,^^17^^,^^29^ Analysis of differentially expressed (DE) miRNAs revealed significantly decreased miR-486-5p expression in the DRG of SNS-gp130^−/−^ compared to control gp130^fl/fl^ (fl) and wild-type (WT) mice (Figure 1A; log median ratio for WT: 0.010, for fl: 0.013, and for knockout [KO]: −0.20; t test followed by Bonferroni correction for multiple comparisons, WT vs. fl *p*_adj_ = 0.74, WT vs. KO *p*_adj_ = 0.021, and fl vs. KO *p*_adj_ = 0.042, *n* = 3 per group). No other miRNAs were found to be significantly differentially expressed after adjustment for multiple comparisons (Figure 1A). To support this finding, we quantified the expression of the two single-stranded miRNAs that derive from the precursor miR-486 (pre-miR-486), namely miR-486-5p derived from the 5′-pre-miR-486 end, which was downregulated in our microarray, as well as miR-486-3p derived from the 3′-pre-miR-486 end, which was not present in the microarray. Quantitative reverse-transcription polymerase chain reaction (RT-qPCR) revealed significantly decreased expression for both miR-486-5p and miR-486-3p in DRG obtained from naive SNS-gp130^−/−^ mice compared to fl/fl littermate controls (Figure 1B; for miR-486-5p: mean ± SEM SNS-gp130^−/−^ 0.731 ± 0.059, gp130^fl/fl^ 1.014 ± 0.056, Mann-Whitney U test *p* = 0.0003, *n* = 12/group [6 males and 6 females]; for miR-486-3p: mean ± SEM SNS-gp130^−/−^ 0.779 ± 0.058, gp130^fl/fl^ 1.020 ± 0.063, Mann-Whitney U test *p* = 0.0068, *n* = 12/group [6 males and 6 females]). Furthermore, we performed chromogenic *in situ* hybridization on DRG sections combined with indirect immune fluorescence microscopy and found miR-486-5p localized in mainly small size DRG neurons, some of which were immunoreactive for the neuropeptide calcitonin gene related peptide (CGRP; Figure 1C). miR-486 is an intragenic miRNA embedded in the cytoskeletal adaptor protein ankyrin 1 (*Ank1*) gene located on chromosome 8 and it is likely that miR-486 and its host gene *Ank1* may derive from a single transcript, indicating a shared promoter.^47^^,^^48^^,^^49^^,^^50^^,^^51^ ANK1 protein was highly expressed in mouse DRG neurons (Figure 1D) but in contrast to miR-486, *Ank1* mRNA expression was not significantly reduced in the SNS-gp130^−/−^ DRG (Figure 1B; for *Ank1* mRNA: mean ± SEM SNS-gp130^−/−^ 0.844 ± 0.061, gp130^fl/fl^ 1.014 ± 0.053, Mann-Whitney U test *p* = 0.0887, *n* = 12/group [6 males and 6 females]). Re-analysis of single-cell RNA sequencing (RNA-seq) data of WT mouse and human DRG revealed that *Ank1* mRNA (for mouse and *ANK1* for human mRNA) was predominantly expressed in neurons (Figure S1A), in all mouse (Figure 1E, top) and human (Figure 1E, bottom) neuronal subtypes.^45^^,^^46^

### No effect of miR-486 depletion on pain-like behaviors or sensorimotor coordination

**Figure 2:**
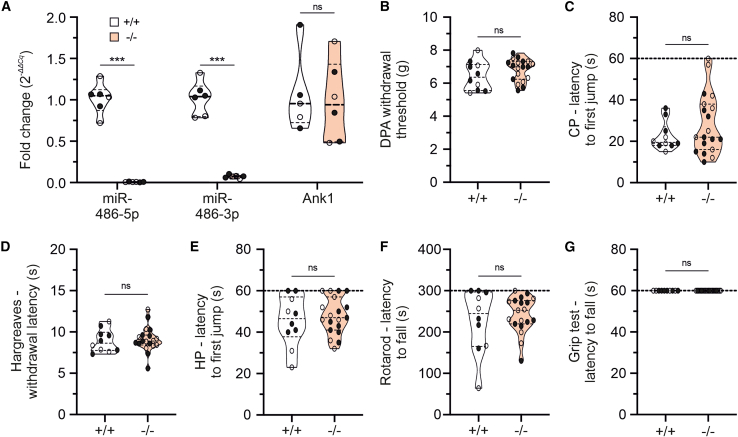
Normal sensitivity to painful stimuli in mice with conditional depletion of miR-486 depletion in sensory neurons (A) miR-486 expression levels are dramatically diminished in miR-486^−/−^ mice, whereas *Ank1* levels remain intact. (B–E) miR-486 deletion is not affecting mechanical (B, dynamic plantar aesthesiometer [DPA] test) and thermal (C, cold plate [CP]; D, Hargreaves; and E, hot plate [HP] tests) hypersensitivity. (F and G) miR-486^−/−^ mice do not exhibit any motor coordination deficits as assessed with the Rotarod test (F) and have similar grip strength to littermate control mice (G). Data are shown as violin plots with medians (solid lines) and interquartile ranges (dotted lines); ∗∗∗*p* < 0.001; ns, not significant.

**Figure 3:**
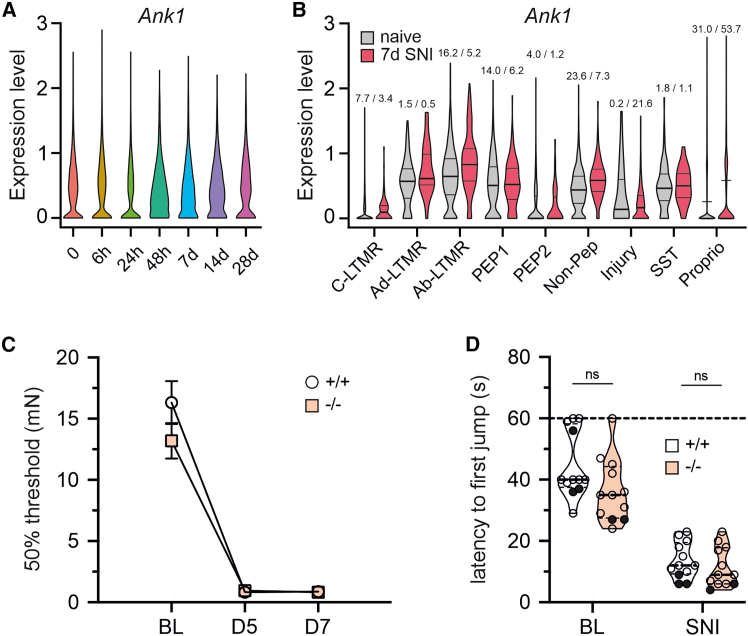
No effect of miR-486 depletion on SNI-induced pain-like behaviors (A) *Ank1* is not significantly upregulated after SNI in neurons and (B) different DRG neuronal subpopulations in the mouse dataset.^45^ C-LTMR, Fam19a4^+^/Th^+^ C-fiber low threshold-mechanoreceptors; Ad-LTMR, Aδ low threshold mechanoreceptors; Ab-LTMR, Aβ low threshold mechanoreceptors; PEP1, Tac1^+/^Gpx3^+^ peptidergic nociceptors; PEP2, Tac1^+^/Hpca^+^ peptidergic neurons; Non-Pep, non-peptidergic neurons; SST, somatostatin positive pruriceptors; Propr, proprioceptors. Numbers above violin plots indicate percentage of cells expressing *Ank1* for mouse DRG neurons. Deletion of miR-486 did not affect SNI-induced pain-like behaviors. (C) Mechanical hypersensitivity measured by von Frey filaments. Data are shown as mean ± SEM. (D) Thermal hypersensitivity assessed by hot plate test. ns, not significant; In panels (A), (B) and (D) data are shown as violin plots with medians (solid lines) and interquartile ranges (dotted lines).

Based on the aforementioned results, we hypothesized that miR-486 downregulation might contribute to deficits observed in SNS-gp130^−/−^ mice.^14^^,^^15^^,^^16^^,^^17^^,^^18^^,^^29^ To address this, we generated transgenic mice with a global depletion of miR-486 and performed sensorimotor phenotyping with standard behavioral tests *in vivo*. miR-486^−/−^ mice did not express the miRNA in DRG, whereas expression levels of *Ank1* were similar to those observed in littermate controls (Figure 2A; for miR-486-5p: mean ± SEM miR-486^+/+^ 1.015 ± 0.076, miR-486^−/−^ 0.007 ± 0.001, Mann-Whitney test *p* = 0.0022, *n* = 6/group [3 males and 3 females]; for miR-486-3p: miR-486^+/+^ 1.017 ± 0.083, miR-486^−/−^ 0.072 ± 0.008, Mann-Whitney U test *p* = 0.0022, *n* = 6/group [3 males and 3 females]; for *Ank1* mRNA: miR-486^+/+^ 1.073 ± 0.219, miR-486^−/−^ 0.984 ± 0.197, Mann-Whitney U test *p* = 0.7922, *n* = 6/group [3 males and 3 females]). Under baseline conditions, withdrawal reflex responses to mechanical as well as thermal stimuli applied to the hind paw were indistinguishable between miR-486^−/−^ and littermate control mice (Figures 2B–2E; Table S1). No differences were observed between genotypes in the rotarod and inverted screen tests indicating normal sensorimotor coordination (Figure 2F; Table S1) and grip strength (Figure 2G; Table S1), respectively, largely excluding a role for miR-486 in nociceptive primary afferents development and function. In the spared nerve injury (SNI) model of neuropathic pain, the average expression levels of neuronal *Ank1* in lumbar DRG L4 and L5 were not significantly altered between naive and injured mice (Figure 3A). Further, differential gene expression analysis for each individual neuronal subtype did not reveal significant changes in *Ank1* expression seven days after injury (Figure 3B). In line with these findings, miR-486 was not dysregulated after SNI (Figure S1B). Furthermore, SNI-induced mechanical and heat hypersensitivity developed in miR-486^−/−^ and littermate control mice in a similar way (Figures 3C and 3D; Table S2). These results suggest that miR-486 did not play a major role in nociception.

### miR-486 is involved in neuronal regeneration

**Figure 4:**
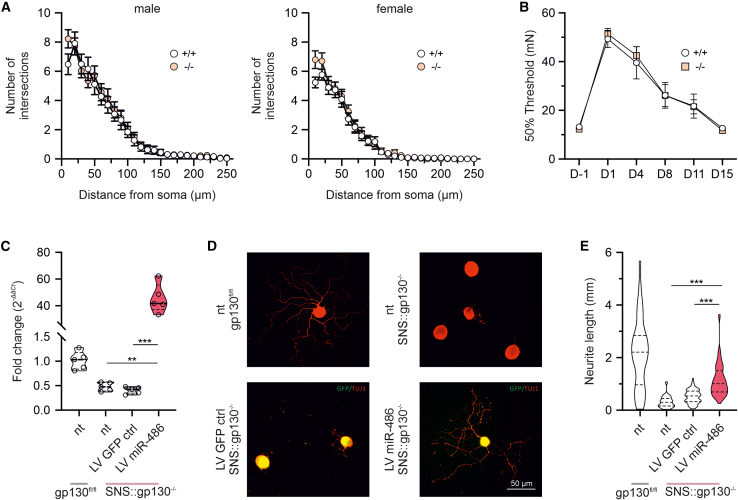
Outgrowth deficit of gp130 deficient neurons is rescued by miR-486 (A) Outgrowth capacity is not affected in DRG neurons derived from miR-486^−/−^ mice (Sholl analysis demonstrating the number of intersections per neurite against the distance from soma [10 μm radius steps] for both sexes and genotypes). (B) miR-486^−/−^ mice recover similarly to littermate controls after sciatic nerve crush (von Frey test); In panels (A) and (B) data are shown as mean ± SEM. (C) Lentiviral overexpression of miR-486 in SNS-gp130^−/−^ DRG neurons induced an increase in neuronal outgrowth capacity. (D) Representative photomicrographs of Tuj-1 staining; scale bar 50 µm. (E) Quantification of total neurite length. nt, non-treated; In panels (C) and (D) data are shown as violin plots with medians (solid lines) and interquartile ranges (dotted lines); ∗∗∗*p* < 0.001 and ∗∗*p* < 0.01.

Neurons from SNS-gp130^−/−^ exhibit a major deficit in neurite outgrowth and regeneration and since miR-486 was the only significantly deregulated miRNA in the DRG of this mice, we hypothesized that miR-486 might be involved in the modulation of neurite outgrowth capacity.^15^ Pathway analysis revealed that miR-486-5p predicted gene transcripts were involved in neuronal processes, including axonogenesis (GO:0007409) and neuron projection (GO:0043005) (Table S3). Next, we assessed neurite outgrowth *in vitro* in DRG neurons derived from miR-486^−/−^ mice and WT littermate controls. Total neurite length and branching points, as well as detailed Sholl analysis showed comparable results for miR-486^−/−^ and control mice (Figures S1C and S1D; Table S4) and differences only emerged between sexes in both genotypes (Figures 4A and S1E; Table S5). Furthermore, IL-6 treatment did not affect neurite outgrowth of miR-486^−/−^ DRG neurons, *in vitro* (DRG neurons treated with IL-6: for KO 979.6 ± 91.16 and for WT 960.0 ± 104.9, Mann-Whitney test *p* = 0.700). Moreover, sensory re-innervation and recovery from hyposensitivity to mechanical stimulation due to partial denervation and subsequent sensory re-innervation after sciatic crush injury *in vivo* was similar in both, miR-486^−/−^ mice and littermate controls (Figure 4B; Table S6). Altogether, the depletion of miR-486 did not generate any deficits in nociceptive processing or regeneration. Therefore, we explored the role of miR-486 in mitigating the regeneration deficit of neurons with a depletion of gp130. As we previously published, DRG neurons from SNS-gp130^−/−^ mice showed significantly reduced neurite outgrowth as compared to control neurons.^15^^,^^17^ miR-486 lentiviral transduction was performed in DRG neuronal cultures obtained from SNS-gp130^−/−^ mice. This resulted in significantly increased miR-486-5p expression (Figure 4C; mean ± SEM: SNS-gp130^−/−^ naive 0.4785 ± 0.395, SNS-gp130^−/−^ control virus 0.4050 ± 0.0322, SNS-gp130^−/−^ miR-486 virus 45.40 ± 4.887; Kruskal-Wallis test *p* = 0.0009; Dunn’s multiple comparisons test: SNS-gp130^−/−^ naive vs. SNS-gp130^−/−^ control virus *p* = 0.8734, SNS-gp130^−/−^ naive vs. SNS-gp130^−/−^ miR-486 virus *p* = 0.0473) and partially rescued neurite outgrowth in DRG neuronal cultures obtained from SNS-gp130^−/−^ mice (Figure 4D; mean ± SEM: gp130^fl/fl^ 2146 ± 212, SNS-gp130^−/−^, naive 322.5 ± 30.92, SNS-gp130^−/−^ control virus 521.8 ± 30.81, SNS-gp130^−/−^ miR-486 virus 1146 ± 73.55; Kruskal-Wallis test *p* < 0.0001; Dunn’s multiple comparisons test: SNS-gp130^−/−^ naive vs. SNS-gp130^−/−^ control virus *p* = 0.0083, SNS-gp130^−/−^ naive vs. SNS-gp130^−/−^ miR-486 virus *p* < 0.0001). Altogether, these results support a specific, context-dependent regulatory role of miR-486 in gp130-dependent neurite outgrowth.

## Discussion

In WT mice, miR-486 was detected in DRG neurons, including peptidergic nociceptors, and identified as the only deregulated miRNA in DRG neurons of mice with a conditional depletion of gp130. Although SNS-gp130^−/−^ mice are hyposensitive to noxious stimuli and show a severe deficit in neuro-regenerative capacities, transgenic mice with a global depletion of miR-486 did not exhibit similar deficits neither in basal sensorimotor and SNI-induced neuropathic pain-like behaviors nor sensory re-innervation after crush injury. In contrast, overexpression of miR-486 in cultured DRG neurons from SNS-gp130^−/−^ partially rescued the defective neurite outgrowth, suggesting a very specific, context-dependent role of this miRNA in gp130-regulated neuro-regenerative processes.

Although miRNAs, including miR-21, miR-431, and miR-511, which are controlled by IL-6 in sensory neurons, contribute to the generation of neuropathic pain,^10^ this does not apply to all deregulated miRNAs after nerve injury. Multiple miRNAs are expressed in the mouse brain, and numerous studies address the possible involvement of individual miRNAs in regulating neurogenesis, brain development, and brain function.^52^^,^^53^ In this context, miR-486, which has not been identified to be regulated by IL-6 in sensory neurons,^10^ is a pleiotropic miRNA that can act as an oncomiR as well as a tumor-suppressor,^54^ and is associated with skeletal muscle development,^55^ coronary and heart disease, cystic fibrosis^54^ as well as neurogenesis.^54^^,^^56^ Deregulation of miR-486 has been associated to mild cognitive impairments,^57^ neurodegenerative disorders,^58^^,^^59^^,^^60^ autism,^61^ and amyotrophic lateral sclerosis.^62^ However, the mechanistic insight into the miR-486 action in the nervous system are not consistent. For example, a recent cell type-specific analysis provides evidence for the involvement of miR-486-5p in brain development by promoting neurogenesis and generation of cortical progenitors,^56^ whereas in adulthood, miR-486 overexpression results in reduced neurogenesis.^63^ miR-486 as cargo of extracellular vesicles improves neurological deficits and reduces the infarct ratio after experimental ischemic brain injury.^64^ In contrast to these reports, which support the idea that miR-486 may have beneficial effects in the nervous system, miR-486 infusion in healthy mice increases neuronal death and deteriorates motor functions.^65^ Intronic miRNAs, such as miR-486 can be co-regulated with their host genes. *Ank1* was slightly downregulated in gp130-depleted sensory neurons (*p* = 0.0887) and this may suggest a potential co-regulation of the two potentially through shared transcriptional mechanisms.^47^^,^^48^^,^^49^^,^^50^^,^^51^ In turn, this could raise the possibility that Ank1 and miR-486 may participate synergistically in injury-induced neuroregenerative processes in the PNS; however, the exact mechanisms need to be explored.

miR-486 has been related to IL-6; however, in this case, its roles are controversially discussed as having anti-inflammatory^66^^,^^67^ as well as pro-inflammatory effects.^68^^,^^69^^,^^70^ Specific miRNAs, but not miR-486, are regulated by IL-6 in DRG and are related to neuropathic pain states.^10^ However, miR-486 is upregulated after spinal cord injury^65^ and associated with intervertebral disc degeneration, one of the main causes of lower back pain.^71^^,^^72^ Interestingly, miR-486 reduces cellular excitability by downregulating hyperpolarization-activated cyclic nucleotide-gated channel 4^73^ and its downregulation might aggravate nociceptor hyperexcitability and ectopic discharges underlying neuropathic pain-like behavior.^74^ Despite the aforementioned points, miR-486^−/−^ mice performed in all behavioral tests in a manner that was indistinguishable from littermate controls, suggesting no major role for miR-486 in acute nociception, injury-induced neuropathic pain-like behaviors in mice as well as peripheral nerve regeneration. Intriguingly, we found that the first seven nucleotides of miR-486 (most of the seed region) have been evolutionarily conserved in our KO mouseline. Therefore, it is possible that mice analyzed in this study produce a novel miRNA, whose sequence partially overlaps with miR-486.

However, miR-486 lentiviral transduction rescued the severely hampered neurite outgrowth of neurons with a depletion of gp130 and, therefore, a very specific role of this miRNA in gp130 mediated neuro-regeneration is more likely. Based on our current findings and the opposing effects of miR-486 in developing vs. adult brain,^56^^,^^63^ we anticipate that low miR-486 expression resulting from depletion of gp130 could be associated with the compromised ability of neurons to regenerate processes. Since expression of the Na_v_1.8 promotor driving gp130 depletion begins around birth, when DRG neuron development is mostly finalized,^28^ major structural deficits are not evident in SNS-gp130^−/−^ mice.^16^ Surprisingly, in miR-486^−/−^ mice, the absence of miR-486 throughout embryonic development did not lead to any major structural or behavioral deficits. Rather than reflecting differences in neurodevelopmental programs between the peripheral vs. central nervous system, the lack of a strong neuronal phenotype suggests that effective mechanisms are in place compensating for the absence of miR-486 during neurodevelopmental processes. For example, other miRNAs, including miR-21, miR-199, miR-26a or miR-222, likewise support neurite outgrowth and regeneration.^75^^,^^76^^,^^77^ Some of these miRNAs, e.g., miR-21 or miR-199, are emerging as critically important regulatory hubs in neurodevelopment that might successfully compensate the lack of miR-486.^78^^,^^79^ Overexpression of miR-486 in gp130-depleted sensory neurons, partially rescued neurite outgrowth, suggesting that this miRNA could act as a modulator of peripheral neuroregeneration in a context dependent manner. While miR-486-5p partially rescued neurite outgrowth in the absence of gp130, the absence of an outgrowth deficit in miR-486-deficient mice indicated that miR-486 is not the sole mediator of gp130-dependent neuronal regeneration and additional regulatory processes are critically involved in peripheral nerve regeneration.

miR-486-5p is highly enriched in extracellular vesicles secreted by specific stem cells, and secreted miR-486-5p improves cutaneous wound healing through its target gene Sp5.^80^ Our results extend on and further support the idea that miR-486 may offer novel options for therapeutic interventions improving neuronal regeneration and reconnection in disorders associated with nerve injury and neuron degeneration resulting from neuropathic disorders.

## Materials and methods

### Animals

Mice were maintained under standard pathogen-free conditions, at 24°C on a 12 h light/dark cycle and had free access to autoclaved food and water. All experiments were performed under the ethical guidelines and animal welfare regulations (Medical University of Innsbruck) as well as the European Communities Council Directive of 22nd September 2010 on the protection of animals used for scientific purposes (2010/63/EU) and all procedures were approved by the Austrian National Animal Experiment Ethics Committee of the Austrian Bundesministerium für Wissenschaft und Forschung (BMWF-66.011/0102-WF/V/3b/2015, 66.011/0128-V/3b/2018, 2021–0.524.223). Mice (8–12 weeks old) of both sexes were used. SNS-gp130^−/−^ and floxed gp130^fl/fl^ mice were bred and genotyped in our animal facility as previously described.^16^

### Generation of global miR-486 knockout mice

Transgenic mouse production was performed by the Gene Editing & Embryology Facility at EMBL, Rome. The Mir-486 KO allele was created by CRISPR-Cas9 editing technology using C57BL/6J (Charles River) zygotes. Briefly, CRISPR crRNA oligo (5′-ACCTCGGGGCAGCTCAGTAC-3′) was annealed with tracrRNA. The target region was Mir-486, ENSMUST00000093576.5, Mir-486-201; genomic coordinate chromosome 8:23,632,571-23,632,698. The annealed single-guided RNA (sgRNA) was complexed with Cas9 protein (Cas9 protein: 50 ng/μL, sgRNA: 20 ng/μL). All CRISPR reagents were synthesized by IDT (Integrated DNA Technologies, Inc., Iowa, USA). Cas9 protein and sgRNA were co-microinjected into zygote pronuclei using standard protocols^81^ and after overnight culture, 2-cell embryos were surgically implanted into the oviduct of day 0.5 *post-coitum* pseudopregnant CD1 mice. The founder mouse had a deletion of 46 base pairs depicted in Figure S2 confirmed via Sanger sequencing. The mutant strain was maintained on a C57BL/6JCrl genetic background. Mutation transmission was confirmed on randomly chosen mice using Sanger sequencing.

Mice were backcrossed with C57BL6/J mice from Janvier labs for at least eight generations before used for experiments and were routinely genotyped (forward primer: 5′-CCAGGCTACGCAACGAAG-3′, reverse primer: 5′-GGGGAAGGGGCAATAAC-3′). Matings were heterozygous to heterozygous mice to obtain litters consisting of KOs and WT littermate controls. Homozygous backcrossed miR-486^−/−^ were viable, indistinguishable from their littermate controls and did not show any obvious signs of dysfunction or behavior deficits in mechanical and heat sensitivity as well as sensorimotor coordination.

### Nerve injury models

The SNI model was utilized to induce peripheral neuropathic pain as previously described.^82^ Briefly, anesthesia was induced via an intraperitoneal injection of 10 mg/kg xylazine and 100 mg/kg ketamine (both from AniMedica GmbH-a LIVISTO company, Senden, Germany). An incision was made on the skin at the lateral surface of the left thigh exposing the sciatic nerve. For mice subjected to the SNI surgery, the tibial and common peroneal nerves were ligated and transected, whereas in sham-operated mice the sciatic nerve was only exposed.^29^^,^^83^^,^^84^ Sciatic nerve crush injury was utilized to study peripheral nerve regeneration. Mice were anesthetized, the sciatic nerve was exposed and crushed perpendicularly, as previously described.^15^

### Behavioral tests

Mechanical sensitivity was assessed before (baseline) and after SNI (days 5 and 7) or crush injury (days 1, 4, 8, 11, and 15) using pre-calibrated von Frey filaments (1.4, 2.8, 4, 8, 16, 22.6, 32, and 45.3 mN) and 50% withdrawal threshold was estimated using the up-and-down method.^85^^,^^86^^,^^87^ Mice were placed in individual chambers made of plexiglass with a wire mesh floor and stimulation was applied on the lateral side of the plantar surface of the left paw. Additionally, for baseline mechanical sensitivity assessment, we used the dynamic plantar aesthesiometer (DPA, Ugo Basile, Gemonio VA, Italy, cat no. 37550) as previously described.^84^ In brief, mice were acclimated to the testing apparatus for 15 min. Ascending force (0–10 g at a rate of 1 g/s and a cut-off of 10 s) was applied to the plantar surface of the left hind paw via a steel rod and latency time to paw withdrawal was automatically recorded. The average of three recordings was subsequently calculated.

Thermal and cold sensitivity was assessed using the hot- and cold-plate tests, respectively. Initially, mice were placed on a 30°C heated plate for 5 min. Subsequently, mice were either placed on a 50°C heated plate (hot-plate) or a 0°C cooled plate (cold-plate). Sessions were recorded and latency for the first jump was analyzed offline. To avoid tissue damage, cutoff was set at the third jump or at 1 min, depending on which criterion would occur first. Thermal sensitivity was further assessed using the Hargreaves test^88^ as previously described.^15^ Mice were habituated to the testing apparatus for 15 min. Infrared intensity was set to 50 on the Hargreaves apparatus (Ugo Basile, cat no. 37570) and the beam was pointed at the plantar surface of the left hind paw, followed by automatic assessment of the withdrawal latency. The average of three recordings was subsequently calculated.

Motor coordination was assessed using the Rotarod test (Ugo Basile, cat no. 47650). One day before the experiment, mice were trained to balance on the cylinder revolving at 4 rpm for 3 min. For the actual experiment, mice were placed on the cylinder, which was rotating at an initial speed of 4 rpm. After positioning all mice, the device was programmed to increase rotational speed from 4 to 40 rpm over the time course of 5 min. Latency to fall was recorded and averaged for three consecutive trials with 15 min intertrial intervals.

Grip strength was evaluated utilizing Kondziela’s inverted screen test.^89^ Mice were placed on top of a 43 × 43 cm wire mesh (12 mm squares of 1 mm diameter wire), which was subsequently rotated 180°. Latency to fall with a cut-off at 1 min was measured and grip strength of both hind paws was manually scored on a scale ranging from 5 (no gripping), 2.5 (moderate grip), to 0 (tight grip), as previously described.^89^

### Tissue collection

Tissue was harvested from SNS-gp130^−/−^, gp130^fl/fl^, miR-486^−/−^, and miR-486^+/+^ mice of both sexes. Mice were anesthetized with isoflurane and decapitated. Lumbar DRG L3-5 were either snap frozen in liquid nitrogen (for gene expression analysis) or incubated in 4% paraformaldehyde (PFA) for 24 h at 4°C, then in 25% sucrose in PBS for another 24 h at 4°C and finally embedded in Tissue Tek (Sakura Finetek) for indirect immunofluorescence microscopy. All samples were stored at −80°C until use. For DRG primary neuronal cultures, tissues were processed as described below (see “DRG primary cultures” section).

### miRCURY locked nucleic acid array miRNA profiling

DRG derived from WT, SNS-gp130^−/−^, and gp130^fl^^/fl^ mice were snap-frozen. RNA was extracted and subjected to miRCURY locked nucleic acid (LNA) array miRNA profiling performed by Exiqon, Denmark. RNA quality was assessed using Bioanalyzer 2100 and Nanodrop instruments. Total RNA (0.37 μg) from sample and reference was labeled with Hy3 and Hy5 fluorescent label, respectively, using the miRCURY LNA Array power labeling kit (Exiqon) following the procedure described by the manufacturer. The Hy3-labeled samples and an Hy5-labeled reference RNA sample were mixed pairwise and hybridized to the miRCURY LNA array version 11.0 (Exiqon), which contains capture probes targeting all miRNAs for human, mouse, or rat registered in the miRBASE version 13.0 at the Sanger Institute. The hybridization was performed according to the miRCURY LNA array manual using a Tecan HS4800 hybridization station (Tecan, Austria). After hybridization, the microarray slides were scanned and stored in an ozone free environment (ozone level below 2.0 ppb) in order to prevent potential bleaching of the fluorescent dyes. The miRCURY LNA array microarray slides were scanned using the Agilent G2565BA Microarray Scanner System (Agilent Technologies, Inc., USA) and the image analysis was carried out using the ImaGene 8.0 software (BioDiscovery, Inc., USA). The quantified signals were background corrected (Normexp with offset value 10^90^) and normalized using the global Lowess (locally weighted scatterplot smoothing) regression algorithm. The microarray data are available from the corresponding authors upon reasonable request.

### DRG primary cultures

DRG primary neuron cultures were prepared as previously described.^14^^,^^91^^,^^92^ Briefly, DRG were cleaned from the connective tissue, treated with liberase, incubated in trypsin, washed, and mechanically dissociated. The mixture was centrifuged in 3.5% BSA (A7906, Sigma), resuspended and neurons were plated on dishes or on coverslips, coated with poly-L-lysine/laminin (P4707/L2020, Sigma), and cultured in TNB-100 medium (F8023, Tico Europe) supplemented with protein-lipid complex (F8820, Tico Europe), L-glutamine (final concentration 0.2 mM, 25030149, Gibco) and 2% penicillin/streptomycin (15140122, Gibco) with 25 ng/mL NGF 2.5S (N-100, Alomone Labs), at 37°C in a humidified atmosphere containing 5% CO_2_.

### Outgrowth assay

Primary DRG neurons were seeded at low density (∼2,000 cells/coverslip) on 12-mm coverslips coated with poly-L-lysine/laminin. Cells were cultured in full TNB-100 medium (protein-lipid complex, L-glutamine, penicillin, and streptomycin) supplemented with 25 ng/mL NGF 2.5S. After 48 h, cells were fixed with 4% PFA (Thermo Fisher Scientific, cat no. J19943-K2) in PBS for 10 min. Neurons were permeabilized in 0.1% Triton in PBS for 3 min, washed twice with PBS for 5 min each. After blocking with 1% BSA in PBS for 30 min, cells were incubated with TuJ-1 mouse primary antibody (R&D Systems, cat no. MAB1195; 1:1,000 in PBS supplemented with 1% BSA) for 1 h. Subsequently, cells were washed twice with PBS and incubated with an anti-mouse, AF-594 fluorophore conjugated secondary antibody (Thermo Fisher Scientific, cat no. A21201; 1:1,000 in PBS) for 30 min. Cells were stained with 4′,6-diamidino-2-phenylindole (DAPI; Thermo Fisher Scientific, cat no. D1306; 1:10,000 in PBS) and washed three times for 5 min with PBS. Coverslips were mounted on microscopy slides using Mowiol 4–88 (Roth, cat no. 0713.2). Imaging was performed with an Axio Imager Z1 microscope (Carl Zeiss) equipped with a cooled charge-coupled device (CCD) camera using a 25×, 0.8 NA oil-immersion objective lens. At least 20 single neurons per mouse without contact with neighboring cells were imaged. Neurite tracing for total neurite length assessment, branching point counting, and Sholl analysis was performed using the simple neurite tracer’s moniker (SNT) application of the image-processing program Fiji.^93^ For Sholl analysis, a series of concentric circles with their centers aligning in the cell soma and radii increasing in 10 μm increments was projected onto individual neurons followed by counting of the neurite intersections per circle.

### Viral vectors

Ready to use viral particles were purchased from Genecopoeia. Lentivirus overexpressing mmu-miR-486 (cat no. LP-MmiR3215-MR03-200-S) and respective control (cat no. CmiR0001-MR03) had a cytomegalovirus (CMV) promoter and an eGFP tag.

### RNA extraction and RT-qPCR

RNA was extracted from DRG collected from SNS-gp130^−/−^, gp130^fl^^/fl^, miR-486^−/−^, miR-486^+/+^, and WT mice of both sexes using peqGOLD TriFast reagent (VWR, 30–2010) according to manufacturer’s instructions. RNA concentrations were measured with Nanodrop 2000 (Thermo Fisher Scientific). All reagents for RT-qPCR were obtained from Thermo Fisher Scientific.

For miRNA expression analysis, reverse transcription and qPCR reactions were performed according to the protocol provided by the supplier (Thermo Fisher Scientific) as previously described.^94^ Briefly, reverse transcription reactions had a final volume of 15 μL and contained: 10 ng of total RNA, 1× reverse transcription buffer, 5.5 mM MgCl_2_ (GeneAmp 10× PCR Buffer II and MgCl_2_, no. N8080130), 1 mM dNTPs, RNase inhibitor (no. N8080119), 50 units of MultiScribe Reverse Transcriptase (no. 4311235), and 1× RT specific primers (see assay IDs in the following text), adjusted with nuclease free water (no. R0582). Reactions were thoroughly mixed and the RT program was as follows: 30 min at 16°C, 30 min at 42°C, 5 min at 85°C. Each qPCR reaction had a final volume of 20 μL and contained the following: 1.33 μL of the RT product, 1× TaqMan Universal Master Mix II, no UNG (no. 44440049), 1× of the appropriate assay (see assay IDs in the following text), and nuclease free water. The miRNA assays used were: hsa-miR-486 (assay ID 001278), hsa-miR-486-3p (assay ID 002093). sno202 (assay ID 001232) and sno429 (assay ID 001240) served as reference genes.

For mRNA expression analysis, reverse transcription reactions had a final volume of 20 μL and contained: total RNA, 1× reverse transcription buffer, 5.5 mM MgCl_2_ (GeneAmp 10× PCR Buffer II and MgCl_2_, no. N8080130), 0.5 mM dNTPs, 8 units Ribolock (no. EO0381), 35 units of MultiScribe Reverse Transcriptase (no. 4311235), and 1× random hexamer primer (no. SO142), adjusted with nuclease free water (no. R0582). Reactions were thoroughly mixed and the RT program was as follows: 10 min at 25°C, 30 min at 48°C, 5 min at 95°C. Each qPCR reaction had a final volume of 20 μL and contained the following: 50 ng of the RT product, 1× TaqMan Universal Master Mix II, no UNG (no. 44440049), 1× of the appropriate assay (see assay IDs in the following text), and nuclease free water. The mRNA assays used were: *Ank1* (assay ID Mm00482889_m1), *Hprt* (assay ID Mm00446968_m1), *Sdha* (assay ID Mm01352363_m1), and *Tfrc* (assay ID Mm00441941_m1). *Hprt*, *Sdha*, and *Tfrc* were used as the reference genes.

For miRNA and mRNA qPCR, reactions for each biological replicate were run in duplicates on optical 96-well reaction plates (4346906) in the QuantStudio 6 Pro Real-Time PCR system with the following parameters: Holding step at 95°C for 10 min, 40 cycles of 15 s at 95°C and finally 1 min at 60°C. Gene expression analysis was performed using the 2^*-ΔΔCq*^ method.

### *In situ* hybridization

*In situ* hybridization was performed on cryostat sections (12 μm) from Zamboni-fixed DRGs.^95^^,^^96^^,^^97^ After washing, acetylation (triethanolamine/HCl/acetic anhydride), and digestion with proteinase K (2 μg/mL), sections were incubated with pre-hybridization buffer. Target miRNAs were hybridized overnight with 0.34 pmol of specific digoxigenin (DIG)-labelled antisense mirCURY probes directed against mmu-miR-486-5p, the reference U6 snRNA or scrambled probes (Exiqon) diluted in hybridization buffer.^97^ Washing with saline-sodium citrate (SSC) buffer was followed by blocking and detection of the DIG labeling using alkaline phosphatase coupled anti-DIG antiserum and 5-bromo-4-chloro-3-indolyl-phosphate/nitro blue tetrazolium (BCIP/NBT; Roche Diagnostics, Castle Hill, Australia).

### Indirect immunofluorescence microscopy

Immunohistochemistry staining was performed on DRG 12 μm thick cryosections. The sections were blocked with 5% BSA in 0.3% Triton X-100 for 1 h at room temperature. Sections were incubated with the primary antibodies overnight at +4°C. For anti-ANK1 primary antibody (Invitrogen, MA5-27723) a 1:100 dilution was used. After washing the slides with PBS, the secondary antibody (goat anti-mouse IgG AF594 1:1,000, Abcam no. ab150116) together with DAPI (1:10,000) was applied for 1 h at room temperature. Slides were rinsed twice and washed two times with PBS. Subsequently, slides were mounted in Mowiol and analyzed using the Zeiss Axiovert 200M fluorescence microscope and MetaView (version 7.8, Molecular Devices).

### *In silico* analysis of DRG single-cell datasets

Publicly available DRG single-cell (GSE155622, mouse^45^) and single-nuclei (GSE168243, human^46^) RNA-seq datasets were downloaded from GeoData and reanalyzed using R (4.2) and Seurat (4.3). Preprocessing per dataset was performed individually and included log-normalization to handle skewed data distribution and additional data integration of the individual batches from the mouse dataset. Cell type annotation in the mouse data was performed following marker genes proposed in,^45^ while the neuronal subtypes were annotated according to marker genes from Renthal et al.^98^

### miRNA target prediction and pathway enrichment analysis

*In silico* target prediction of mmu-miR-486-5p was performed using the DIANA-microT webserver and microRNA::mRNA interactions with a microT interaction score ≥0.7 were queried.^99^ Gene Ontology (GO) pathway enrichment analysis was performed using the g:Profiler R package *gprofiler2*,^100^ with default g:SCS (set counts and sizes) multiple-testing correction (significance threshold *p* < 0.05). To eliminate redundant GO terms, a best-per-parent filter was implemented in R by removing any enriched term that was an ancestor of another term in the result set, thereby retaining only the most specific term in each GO branch.

### Statistical analyses

The generalized linear mixed model used for analyzing the Sholl analysis was fitted in R (version 4.1.1; R Core Team 2021) using the function glmer of the package lme4 (version 1.1–27.1). All other statistical analyses as well as figure preparation were performed using GraphPad Prism version 10.2.0. The collected data were analyzed using appropriate parametric and non-parametric statistical tests, depending on data distribution (two-tailed t test or Mann-Whitney U test for two group comparisons, ANOVA or Kruskal-Wallis H-test for comparisons of more than two groups). Appropriate tests correcting for multiple comparisons were applied. The criterion for statistical significance was set to *p* < 0.05.

## Data availability

All data are maintained on institutionally managed servers and are available upon request. *In silico* analysis scripts are publicly available (https://github.com/ZiDa20/mir486_paper).

## Acknowledgments

We thank Kathrin Braun and Federica Vercelli for expert technical assistance. This research was funded in part by the 10.13039/501100002428Austrian Science Fund (FWF) (Grant-DOI: https://doi.org/10.55776/P28611 to M.K. and Grant-DOI: https://doi.org/10.55776/P36229 to T.K.) and by the European Commission under FP7: GA no. 602133 (coordinator M.K.). For open access purposes, the author has applied a CC BY public copyright license to any author accepted manuscript version arising from this submission.

## Author contributions

Conceptualization, T.K., K.K., and M.K.; data curation, T.K., K.K., V.H., D.Z., M.P., S.Q., N.M., and R.V.H.; formal analysis, T.K., K.K., V.H., D.Z., M.P., S.Q., N.M., and R.V.H.; funding acquisition, T.K. and M.K.; investigation, T.K., V.H., M.P., S.Q., N.M., L.C., P.A.H., and R.V.H.; methodology, T.K., K.K., D.Z., M.P., L.C., P.A.H., and M.K.; project administration, T.K. and M.K.; software, D.Z.; resources, T.K., D.Z., L.C., P.A.H., H.S., and M.K.; validation, T.K., K.K., and D.Z.; visualization, T.K., K.K., V.H., and D.Z.; writing – original draft, T.K. and M.K.; writing – review & editing, T.K., K.K., V.H., D.Z., M.P. S.Q., N.M., L.C., P.A.H., R.V.H., H.S., and M.K.; supervision, T.K. and M.K. All authors read and approved the final version of the manuscript.

## Declaration of interests

The authors declare no competing interests.

## Supplemental information

Document S1. Figures S1 and S2 and Tables S1–S6

Document S2. Article plus supplemental information
