# Supplementary material for: Context dependent role of miR-486 promoting neuroregeneration of primary sensory neurons downstream of interleukin-6 signal transducer
Source: Mol Ther Nucleic Acids. 2025 Aug 6;36(3):102670. doi: 10.1016/j.omtn.2025.102670 (PMC12398936; doi:10.1016/j.omtn.2025.102670)
Supplement: Document S1. Figures S1 and S2 and Tables S1–S6 [file mmc1.pdf]

OMTN, Volume *36*

Supplemental information

Context dependent role of miR-486 promoting neuroregeneration of primary sensory neurons downstream of interleukin-6 signal transducer

Theodora Kalpachidou, Kai Kummer, Valentina Handle, David Zimmermann, Maria Peteinareli, Serena Quarta, Natalia Mach, Laura Castaldi, Paul A. Heppenstall, Rainer V. Haberberger, Hermona Soreq, and Michaela Kress

Supplemental Figures

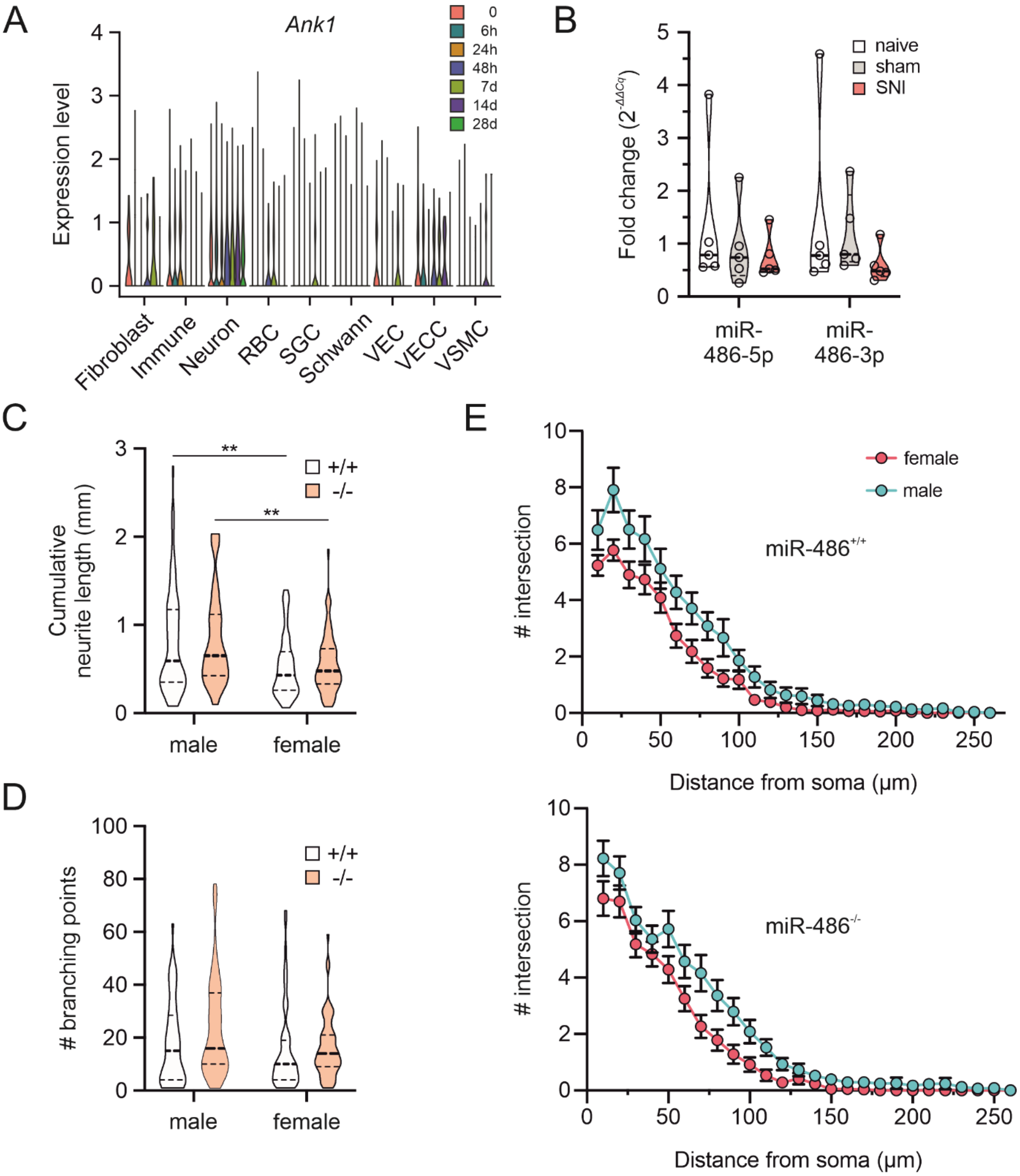

Figure S1: A. Comparison of *Ank1* expression between all DRG cell types and regulation post SNI in the mouse dataset;[1] RBC: Red Blood Cells, SGC: Satellite Glia Cells, VEC: Vascular Endothelial Cells, VECC: Vascular Endothelial Capillary Cells, VSMC: Vascular Smooth Muscle Cells. B. miR-486 is not regulated seven days post SNI. *In vitro* outgrowth assay of DRG neurons derived from miR-486$^{+/+}$ and miR-486$^{-/-}$ mice: C. Cumulative neurite length in µm for male and female miR-486$^{+/+}$ and miR-486$^{-/-}$ mice, revealed a sex difference consistent for both genotypes. D. Number of branch points in

male and female mice. E. Sholl analysis demonstrating the number of intersections per neurite against the distance from soma (10µm radius steps) for both sexes and genotypes. **p<0.01.

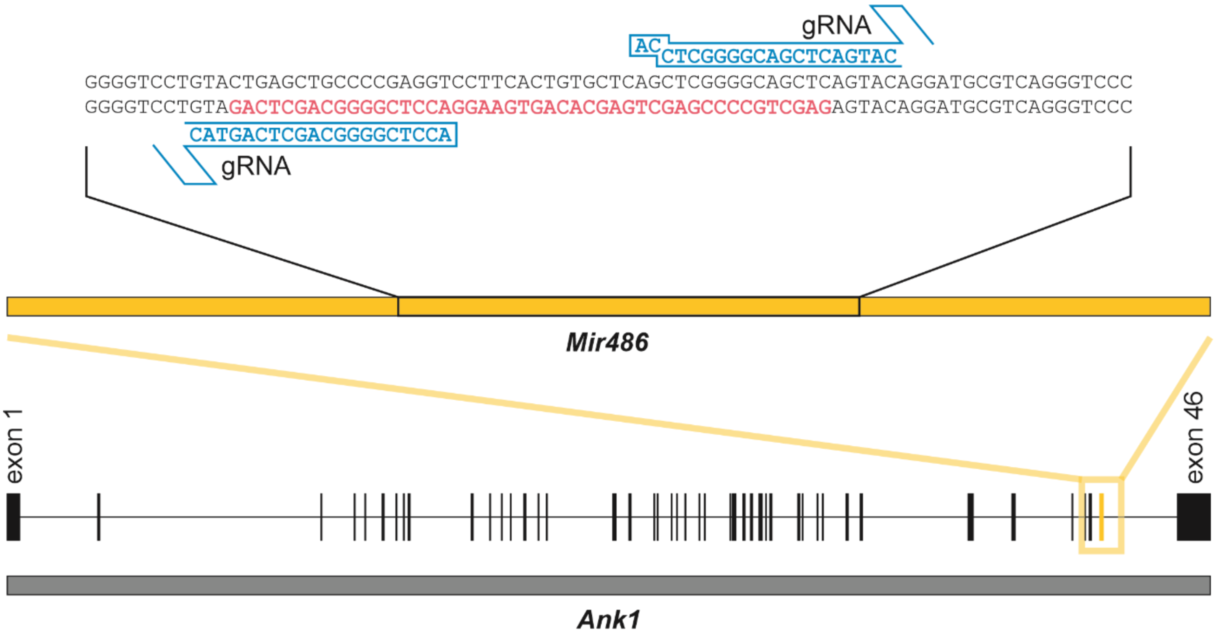

Figure S2: Schematic representation depicting the location of MiR486 on the Ank1 host gene (bottom). Target positions of guide RNAs (gRNA in blue, up) for CRISPR-mediated 46 bp deletion (pink) resulting in MiR486 knockout.

Supplemental Tables

Table S1: Descriptive statistics and statistical tests employed to analyze baseline behavioral tests performed on miR-486 ko and littermate controls

		Mean±SEM						n numbers			
								$miR-486^{+/+}$		$miR-486^{-/-}$	
Figure	Behavioral test	$miR-486^{+/+}$	$miR-486^{-/-}$	Unit	Statistical test	p-value	p-value summary	♂	♀	♂	♀
2B	DPA	6.461±0.277	6.829±0.152	g	Mann-Whitney, 2-tailed	0.206	ns	4	6	9	10
2C	CP	22.5±2.177	28.42±3.360	s	Mann-Whitney, 2-tailed	0.383	ns	4	6	9	10
2D	Hargreaves	8.923±0.431	9.115±0.351	s	Mann-Whitney, 2-tailed	0.644	ns	4	6	9	10
2E	HP	45.40±3.848	47.89±2.036	s	Mann-Whitney, 2-tailed	0.642	ns	4	6	9	10
2F	Rotarod	227.6±24.50	242.3±10.04	s	Mann-Whitney, 2-tailed	0.946	ns	4	6	9	10
2G	Grip test	60.00±0.00	60.00±0.00	s	Mann-Whitney, 2-tailed	>0.999	ns	4	6	9	10

CP: cold plate
DPA: dynamic plantar aestesiometer
HP: hot plate
SEM: standard error of the mean

Table S2: Descriptive statistics and statistical tests employed to analyze behavioral tests performed on miR-486 ko and littermate controls subjected to SNI

		Mean±SEM										n numbers			
		miR-486$^{+/+}$			miR-486$^{-/-}$							miR-486$^{+/+}$		miR-486$^{-/-}$	
Figure	Behavioral test	baseline	day 5 post SNI	day 7 post SNI	baseline	day 5 post SNI	day 7 post SNI	Unit	statistical test	Results	p-value summary	♂	♀	♂	♀
3C	von Frey	16.51 ±1.768	1.000 ±0.000	1.048 ±0.048	13.38 ±1.454	1.138 ±0.139	1.000 ±0.000	mN	2-way RM ANOVA	surgery x genotype F(2, 44) = 1.935, p=0.1566	ns	8	3	10	3
										surgery F(2,44)=148.0, p<0.0001	****				
										genotype F(1,22)=1.824, p=0.1905	ns				
3D	HP	44.67 ±3.141	n/a	13.67 ±1.703	33.92 ±3.780	n/a	12.15 ±1.863	s	2-way RM ANOVA	surgery x genotype F(1,22) = 1.993, p=0.172	ns	9	3	9	3
										surgery F(1,22)=179.0, p<0.0001	****				
										genotype F(1,22)=3.291, p=0.083	ns				

HP: hot plate

RM: repeated measures

SEM: standard error of the mean

SNI: spared nerve injury

Table S3: Pathway enrichment for predicted targets of mmu-miR-486-5p

Category	term ID	term description	intersection size	term size	adjusted p-value
GO Biological Process	GO:0045944	positive regulation of transcription by RNA polymerase II	53	1332	1.57E-09
GO Biological Process	GO:0048523	negative regulation of cellular process	117	5732	4.55E-04
GO Biological Process	GO:0141124	intracellular signaling cassette	49	1806	2.90E-03
GO Biological Process	GO:0007610	behavior	28	777	4.07E-03
GO Biological Process	GO:0006810	transport	92	4387	5.29E-03
GO Biological Process	GO:0007409	axonogenesis	20	478	1.47E-02
GO Biological Process	GO:0007416	synapse assembly	13	217	1.52E-02
GO Biological Process	GO:0000122	negative regulation of transcription by RNA polymerase II	32	1034	1.80E-02
GO Biological Process	GO:0048638	regulation of developmental growth	17	372	2.34E-02
GO Biological Process	GO:0021766	hippocampus development	9	107	2.76E-02
GO Biological Process	GO:0044772	mitotic cell cycle phase transition	18	423	3.39E-02
GO Biological Process	GO:0060039	pericardium development	5	25	3.63E-02
GO Biological Process	GO:0009968	negative regulation of signal transduction	37	1330	3.74E-02
GO Biological Process	GO:0007169	cell surface receptor protein tyrosine kinase signaling pathway	22	602	4.25E-02
GO Biological Process	GO:0061351	neural precursor cell proliferation	12	206	4.52E-02
GO Cellular Component	GO:0043005	neuron projection	44	1528	4.89E-04
GO Cellular Component	GO:0016020	membrane	179	10237	1.77E-03
GO Cellular Component	GO:0005634	nucleus	176	10122	3.43E-03
GO Cellular Component	GO:0045211	postsynaptic membrane	16	332	3.56E-03
GO Cellular Component	GO:0036477	somatodendritic compartment	33	1109	5.44E-03
GO Cellular Component	GO:0099572	postsynaptic specialization	17	429	2.27E-02
GO Molecular Function	GO:0000978	RNA polymerase II cis-regulatory region sequence-specific DNA binding	65	1090	6.96E-20
GO Molecular Function	GO:0046872	metal ion binding	120	4235	1.55E-11
GO Molecular Function	GO:0001228	DNA-binding transcription activator activity, RNA polymerase II-specific	34	519	1.31E-10
GO Molecular Function	GO:0001227	DNA-binding transcription repressor activity, RNA polymerase II-specific	18	353	2.28E-03
GO Molecular Function	GO:0008134	transcription factor binding	24	653	1.63E-02

Table S4: Descriptive statistics and statistical tests employed for outgrowth assay (neurite length and branching points) of DRG derived from miR-486 ko and littermate controls

Figure	Parameter	Descriptive statistics (mean ± SEM)				Statistical test	Results	p-value summary	Tukey's multiple comparisons test
		male +/+ (*n* = 56)	male -/- (*n* = 61)	female +/+ (*n* = 59)	female -/- (*n* = 60)				
S1D	Neurite length (µm)	812.1 ±80.80	841.5 ±68.37	527.4 ±45.53	560.6 ±43.96	two-way ANOVA	surgery x genotype F(1, 232) = 0.001, p=0.9750<br>sex F(1,232)=21.39, p<0.0001<br>genotype F(1,232)=0.2621, p=0.6092	ns<br>****	males +/+ vs males -/- : p=0.9866<br>**males +/+ vs females +/+ : p=0.0072**<br>males +/+ vs females -/- : p=0.0222<br>males -/- vs females +/+ : p=0.0017<br>**males -/- vs females -/- : p= 0.0063**<br>females +/+ vs females -/- : p=0.9804
S1E	Branching points (#)	17.96 ±2.100	23.33 ±2.309	15.39 ±2.094	16.35 ±1.519	two-way ANOVA	surgery x genotype F(1, 232) = 1.178, p=0.2789<br>sex F(1,232)=5.543, p=0.0194<br>genotype F(1,232)=0.2429, p=0.1204	ns<br>*<br>ns	males +/+ vs males -/- : p=0.2479<br>males +/+ vs females +/+ : p=0.8121<br>males +/+ vs females -/- : p=0.9443<br>males -/- vs females +/+ : p=0.0289<br>males -/- vs females -/- : p= 0.0683<br>females +/+ vs females -/- : p=0.9869

SEM: standard error of the mean

Table S5: Descriptive statistics and statistical tests employed for outgrowth assay (Sholl analysis) of DRG derived from miR-486 ko and littermate controls

		**intersections_transformed**		
Figure	Predictors	Estimates	CI	p-value
4A and S1F	genotype [wt]	-0.04	-0.31 – 0.22	0.743
	sex [male]	0.24	-0.03 – 0.50	0.077
	radius scaled	-0.29	-0.32 – -0.25	**<0.001**
	Observations	2036		
	Marginal R^2 / Conditional R^2	0.117 / 0.214		

Table S6: Descriptive statistics and statistical tests employed for von Frey test applied on miR-486 ko and littermate controls subjected to crush injury

		Mean±SEM																n numbers			
		miR-486$^{+/+}$						miR-486$^{-/-}$										miR-486$^{+/+}$		miR-486$^{-/-}$	
		day post crush injury																			
Figure	Behavioral test	-1	1	4	8	11	15	-1	1	4	8	11	15	Unit	statistical test	Results	p-value summary	♂	♀	♂	♀
4B	von Frey	13.25 ± 0.854	49.33 ± 3.565	39.53 ± 6.657	26.18 ± 5.306	21.75 ± 4.964	12.7 ± 0.667	12.26 ± 0.588	51.34 ± 2.314	42.54 ± 3.599	26.08 ± 4.493	21.44 ± 2.892	11.74 ± 1.179	mN	2-way RM ANOVA	surgery x genotype $F(5,85) = 0.127$, $p=0.986$	ns	4	4	6	5
																surgery $F(5,85)=43.52$, $p<0.0001$	****				
																genotype $F(1,17)=0.032$, $p=0.8604$	ns				

RM: repeated measures

SEM: standard error of the mean

References

1. Wang, K, Wang, S, Chen, Y, Wu, D, Hu, X, Lu, Y, Wang, L, Bao, L, Li, C, and Zhang, X (2021). Single-cell transcriptomic analysis of somatosensory neurons uncovers temporal development of neuropathic pain. Cell Res *31*: 904-918.
